# Functionalized Solid Electrodes for Electrochemical Biosensing of Purine Nucleobases and Their Analogues: A Review

**DOI:** 10.3390/s150101564

**Published:** 2015-01-14

**Authors:** Vimal Kumar Sharma, Frantisek Jelen, Libuse Trnkova

**Affiliations:** 1 Department of Chemistry, Faculty of Science, Masaryk University, Kamenice 5, CZ–625 00 Brno, Czech Republic; E-Mail: vimal.shrma@gmail.com; 2 Institute of Biophysics of the Academy of Sciences of the Czech Republic, V.V.I., Kralovopolska 135, CZ–612 65 Brno, Czech Republic; E-Mail: jelen@ibp.cz; 3 SIX Research Centre, University of Technology Brno, Technicka 3058/10, CZ–616 00 Brno, Czech Republic

**Keywords:** purine derivatives, electrochemistry of purines, carbon electrode, gold electrode, platinum electrode, indium tin oxide electrode, boron doped electrode, modified electrodes

## Abstract

Interest in electrochemical analysis of purine nucleobases and few other important purine derivatives has been growing rapidly. Over the period of the past decade, the design of electrochemical biosensors has been focused on achieving high sensitivity and efficiency. The range of existing electrochemical methods with carbon electrode displays the highest rate in the development of biosensors. Moreover, modification of electrode surfaces based on nanomaterials is frequently used due to their extraordinary conductivity and surface to volume ratio. Different strategies for modifying electrode surfaces facilitate electron transport between the electrode surface and biomolecules, including DNA, oligonucleotides and their components. This review aims to summarize recent developments in the electrochemical analysis of purine derivatives, as well as discuss different applications.

## 1. Introduction

**Figure 1.**
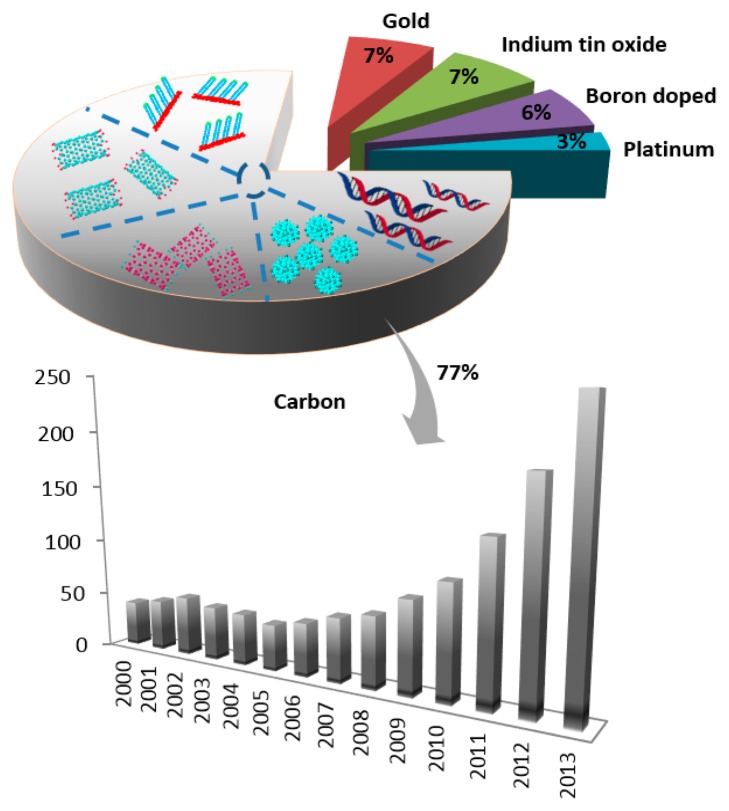
Papers published on electrochemistry of purine derivatives between 2004 and 2013 (from Web of Science database).

Based on their sensitivity, affinity and selectivity biosensors have attracted a broad research interest. Deoxyribonucleic acid (DNA) stores genetic information and is important for protein biosynthesis. Purines are the building blocks of DNA and play an important role in storing genetic information as well as contributing in cell signaling processes. Purine nucleobases (adenine, guanine) and some of their derivatives, *i.e.*, xanthine and hypoxanthine, play an important role in biological processes [1,2]. Purines are widely involved in various diseases, most of them possess great significance in clinical and medical practice, and thus their presence and amount in biological fluids and pharmaceuticals must be carefully identified and quantified. The redox activity of the majority of purines allows their determination by means of sensitive, inexpensive and simple electrochemical sensors. The number of publications in this area is growing and interest in electrochemical analysis of purines in the last decade is documented in Figure 1.

Electrochemical bioanalysis basically contains three components: the biomolecules that recognize the analytes present in the sample, the detector system which transforms the signal resulting from the interaction of these biomolecules, and finally the screen which displays the information in some user-friendly manner. Carbon-based electrodes have been used extensively for the detection of biomolecules during recent years, as shown in Figure 1.

Due to the significant role of carbon-based electrodes in biosensor fabrication, they are widely used and carbon has become a primary electrode material for improving the performance of the biosensor. Surface modifications of electrode by using nano-based materials which exhibit extraordinary electronic and physical properties provide a crucial way of influencing the performance of the biosensor. Various nanomaterials, including nanoparticles, nanotubes and graphene have achieved high versatility as electrode materials.

Earlier studies were frequently done on mercury, mercury-film, or amalgam electrodes utilizing their reduction behavior. The analysis methods connected with the mercury electrode, even if they have advantages in some cases, are not widely used nowadays and therefore not discussed in this review. Our attempt is rather focused on oxidation of the mentioned purine derivatives on solid or solid-modified electrodes. It is known that nucleobases, as parts of DNA, RNA, nucleosides and nucleotides, are electrochemically active in the sense of electron exchange. Some of them can be reduced at negative potentials [3,4], all of them can be oxidized at solid electrodes [5,6]. On the other hand, individual electrochemical investigations including structural or analytical analysis of nucleic acids are frequently based on the electrochemical behavior of their nucleobases. This review is aimed at their electrochemical analysis, which is predominantly based on their oxidation behavior on solid electrodes in water solution by sweep voltammetric methods (linear or cyclic) or pulse voltammetric methods (differential or square-wave) and/or in connection of these methods with stripping techniques or a specific electrode modification which results in more sensitive and selective determination [4,7,8]. Regarding the analysis of adenine and xanthine, their determinations connected with enzymatic reactions, taking place either in solution or at the electrode surface, are not included in this review. Additionally, in case of adenine, the same is also true about the determinations connected with ADP and ATP.

## 2. Electrochemical Oxidation of Purine Derivatives

### 2.1. Adenine and Its Derivatives

The adenine oxidation at carbon electrodes has been intensively studied and discussed by many authors, e.g., [9–11]. The whole process is irreversible and requires the exchange of six electrons and six protons in three subsequent steps (E1, E2, E3) involving formation of 2-hydroxyadenine (6-amino-1,3-dihydro-2*H*-purin-2-one) or its tautomer, then 2, 8-dihydroxyadenine (6-amino-7,9-dihydro-1H-purine-2,8-dione) or its tautomer and diimine species, which can hydrolyze and give pH-dependent products [10–13].

**Scheme 1.**
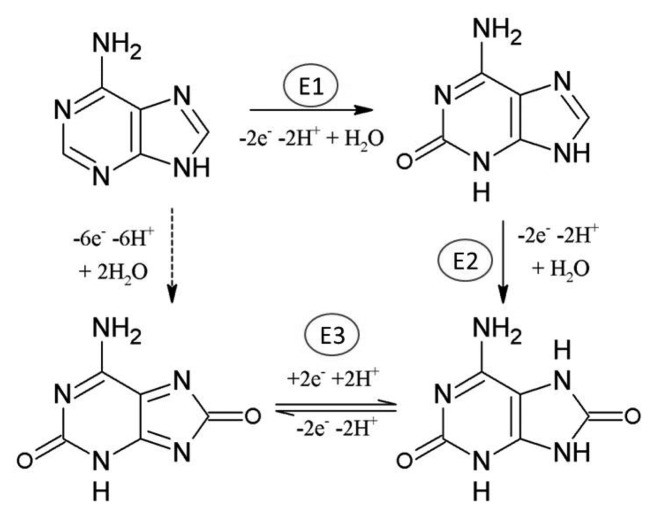
Electro-oxidation of adenine.

The basic scheme is depicted in Scheme 1 [10,11]. The formation of adsorbed dimers was also observed using a carbon fiber microelectrode [14]. It was stated that the oxidation of adenine (Ade) is strongly dependent upon the electrode used and reflects both the kinetics of oxidation and the strength of bonding to the electrode surface [11]. The electrochemical oxidation of adenosine (Ado) [15] and adenosine monophosphate (AMP) [16] is more complex compared to the oxidation of Ade. Ado was found to be oxidized at more positive potentials; at pH 7 the shift is about 300 mV and oxygen-oxygen linked dimers are formed as a major product of oxidation at neutral medium. For AMP the oxidation proceeds in two parallel routes resulting in the formation of a variety of oxygen-oxygen and carbon-carbon linked dimers [16]. Various results dealing with the analysis of Ade and Ado depending on the type of electrode with possible modifications, methods, their detection limit and related applications are summarized in Table 1.

### 2.2. Guanine and Its Derivatives

**Scheme 2.**
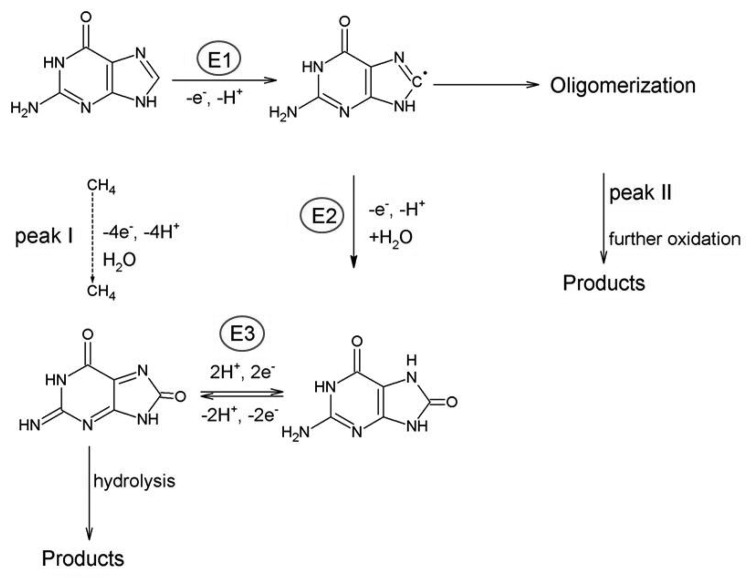
Two possible paths for the electrooxidation of guanine.

The electrochemical oxidation of guanine (Gua) was studied in aqueous media at various carbon electrodes and it was found that voltammetric behavior varied significantly depending on the type of carbon electrode [17] It was also stated that there are two dominating and controlling factors present: (a) the density of basal plane sites on which Gua can adsorb and (b) the density of edge plane sites necessary for the electrooxidation of the analytes. The oxidation mechanism of Gua is a complex one involving exchange of four electrons and four protons (E1, E2, E3). The Gua oxidation proceeds via a two electron oxidation followed by a chemical step to form 8-oxoguanine (2-amino-7,9-dihydro-1*H*-purine-6,8-dione), then 8-oxoguanine is further oxidized to form nonelectroactive products [17] (Scheme 2).

It was also proved that the electrochemical oxidation mechanism of Gua using a carbon fiber microelectrode formation of strongly adsorbed dimers is observed on the electrode surface [14]. Various studies on Gua and Guo with different modification on electrode surface are summarized in Table 2.

#### Xanthine and Its Derivatives

**Scheme 3.**
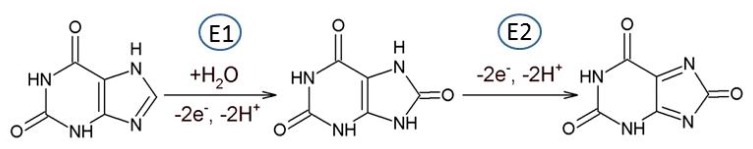
Electrooxidation of xanthine.

The mechanism of xanthine (Xan) oxidation was suggested earlier by several electrochemists [9,56,57], revised and discussed later [58]. The whole process proceeds in two steps (E1, E2); in the first step oxidation to uric acid (7,9-dihydro-1*H*-purine-2,6,8(3*H*)-trione) takes place, which is immediately oxidized to diimine, which is hydrolyzed and gives pH-dependent products, similarly as in the case of Ade. Therefore, the voltammetric signal corresponds to the overall requirement of four electrons and four protons (Scheme 3). The results published on Xan and its derivatives are summarized in Table 3.

## 3. Nucleobase Assemblies on Solid Electrode

### 3.1. Bare Electrode

In 2004, an important paper was published [5], where bare GCE was used for simultaneous determination of all nucleobases in combination with the DPV method. The GCE was not modified, only electrochemical preconditioning including washing, sonication and anodic polarization was done. It was concluded that detection limits in the nano- and micromolar range were obtained for purine and pyrimidine bases and that the results presented show for the first time that the pyrimidine nucleosides and nucleotides are electroactive on GCE and that, in addition to the easy detection of purines, it was also possible to detect simultaneously the oxidation of pyrimidine residues in ssDNA [5]. Bare GCE was also used for the formation of a dsDNA-liposome complex by the decrease of the dsDNA oxidation peaks, dependent upon the incubation time. This behavior was explained considering the electroactive centers of dsDNA, GMP and AMP residues, part of them hidden inside the dsDNA-liposome complex structure and thus unable to reach the GC electrode and preventing their oxidation [39]. Carbon SPE has been investigated as a possible sensor to identify γ-irradiation induced oxidative damage in dsDNA by CV and DPV [34]. The same electrodes were used for a simple differential pulse voltammetric genotoxicity assay utilizing dsDNA in free solution based on the DNA damage by potassium dichromate [27]. Also other types of unmodified electrodes like the pyrolytic graphite electrode (PyGE) and pencil graphite electrode (PeGE) were used for electrochemical monitoring of nucleobases or DNA. DPV by using renewable PeGE was employed to monitor the DNA interaction of Pd(II) and Pt(II) metal complexes with fish sperm dsDNA based on the oxidation signals of Gua and Ade at the surface or in solution [26].

A simple and rapid approach for detecting apurinic sites in DNA, based on direct stripping chronopotentiometric measurements of Ade and Gua nucleobases at a PeGE was described [35]. Experiments with mixtures of free nucleobases and purine nucleosides revealed that the larger A/G ratio observed in the electrochemical analysis of apurinic site-containing oligomers is attributed to the influence of the acid and/or thermal decomposition products. This study represented the first step in developing a simple and direct electrochemical assay of apurinic sites in ssDNA. PyGE represents a good tool for the study of electrochemical oxidation of Ado in a wide range of pH [15] and the electrochemical oxidation mechanism of Ade which included a reflection of both the kinetics of oxidation and the strength of bonding to the electrode surface, both of which are surface specific [11].

### 3.2. Boron-Doped Diamond Electrodes

Boron-doped diamond (BDD) electrodes are attractive for electrochemical analysis due to their advantages, *i.e.*, a large potential window in aqueous solution, a low background current, and a high electrochemical stability. BDD electrodes have been used for the electrochemical oxidation of underivatized-nucleic acids in terms of single stranded and double stranded DNA in connection with cyclic voltammetry and square wave voltammetry [38]. At the BDD electrode, at least two well-defined voltammetric peaks were observed for both single stranded and double stranded DNA. BDD electrode is the first material to show a well-defined voltammetric peak for Ade group oxidation directly in the helix structure of nucleic acid due to its wide potential window. For single stranded DNA, a third peak was observed, related to the pyrimidine group oxidation. Linear calibration results have shown linearity of current with concentrations in the range of 0.1–8 μg/mL for both Gua and Ade residues at as-deposited BDD. Detection limits (S/N = 3) of 3.7 and 10 ng/mL for Ade and Gua residue in single stranded DNA, respectively, and 5.2 and 10 ng/mL for Ade and Gua residue in double stranded DNA, respectively, were observed [38].

BDD electrodes are frequently used in chromatographic or electrophoretic detectors due to their high stability for analysis of biological samples. In the case of simultaneous detection of purine and pyrimidine bases in mild acidic media by using HPLC with amperometric detection no deactivation of the electrode was found during cyclic voltammetric and HPLC measurements [69]. Similarly, a good stability and reproducibility reflecting the minimal adsorption of purines at the BDD surface at microchip capillary electrophoresis coupled with a BDD electrode was observed for the separation and detection of several purines and purine-containing compounds [51]. Quite recently, a BDD electrode was proposed as a simple electrochemical sensor for sensitive determination of Ade and Gua in native, thermally and acid denatured DNA samples from fish sperm and acid denatured DNA samples from human placenta [19]. A boron-doped carbon nanotubes modified glassy carbon electrode was predicted to provide a potential application for the electrochemical detection of DNA in the field of genetic-disease diagnosis [70]. This prediction was made on the excellent results in which the remarkable electrocatalytic activity towards the oxidation of purine bases and direct oxidation of pyrimidine bases was followed at this modified electrode.

A BDD electrode was also used for hydrolyzed oligodeoxynucleotides and DNA, and a report on copper-enhanced label-free anodic stripping detection of Ade and Gua bases in acid-hydrolyzed DNA was published [71]. The analysis was performed in a small volume (50 μL) and efficient stirring during the accumulation step by streaming of an inert gas was used. The accelerated mass transport due to the solution motion resulted in an enhancement of the guanine oxidation signal by about two orders of magnitude (compared to accumulation of the analyte from a solution not containing copper), allowing an easy detection of ∼25 fmol of the ODNs [71]. The proposed technique was shown to be suitable for the determination of purine (particularly guanine) content in DNA samples.

### 3.3. Indium-Tin Oxide (ITO) Electrodes

Indium tin oxide (tin-doped indium oxide) is a solid solution of indium(III) oxide (In_2_O_3_) and tin(IV) oxide (SnO_2_), typically 9:1 by weight, which is optically transparent, electrically conductive, colorless in thin layers, and can be used as a working electrode in electrochemical experiments, e.g., [72]. Its special properties made it a suitable and significant tool for systems dealing with detection of DNA hybridization by electrocatalytic oxidation of Gua [73–75] in the presence of ruthenium tris (2,2′-bipyridine) cation used as a redox mediator for the catalytic reaction [76–79]. ITO surface frequently serves as a substrate for additional modification by different substances, e.g., gold nanoparticles [80], chitosan in combination of MWCNT [81] or graphene [82], titanium dioxide [83], *etc.* An ITO electrode modified by nanogold was used for the simultaneous determination of ADO and ATP with detection limits as 0.07 mM and 0.10 mM, respectively [80]. On the other hand, a composite film, which contains MWCNT along with the incorporation of poly(neofuchsin) [84], has been synthesized also on ITO electrode by potentiostatic methods. This composite electrode was used for the simultaneous determination of Gua and Ade [84] with satisfactory results.

## 4. Surface Modification Approaches to Enhance Electrochemical Activity of Purines

### 4.1. Nanostructured Materials

Nanostructured materials have received widespread considerable attention due to their extraordinary physical and chemical properties. In recent years nanomaterials with tailored morphological features and exclusive potential properties have provoked an immense interest in the range of fields including electrochemical biosensing. Electrochemical sensing significantly exploited nano-based materials as functional electrode materials in order to achieve a simple and inexpensive analytical method for the detection of biomolecules. To understand the electrochemical behavior nanomaterials including graphene, carbon nanotubes (CNTs) and nanoparticles (NPs) have been discussed in this section. Some papers demonstrate an enhanced electrochemical signal on pencil electrode that can be achieved by various nano-based modifications. The unique properties of nano-based materials accelerate electron transfer and enhance the electrochemical activity of biomolecules.

#### 4.1.1. Graphene

Carbon materials have been widely used in electrochemical sensing because of their promising properties, including a wide potential range compared to other solid electrodes, low cost, low background and chemical inertness for biosensing in various electrolyte solutions [85,86]. In 2004 Novoselov *et al.* [87,88] observed that carbon sheets of a thickness of up to few atomic layers possessed electric field effects. These two dimensional (2D) sheets of sp^2^ bonded carbon possess uncommon electronic and electrochemical properties [89–91] making it a promising candidate for electrochemical applications. There has been a significant increase in the use of graphene for electrochemical application in past years. It has been demonstrated that graphene can exhibit potential advantages including electrochemical activity, surface area compared to the other carbon materials including CNTs [92–94] or glassy carbon [95].

**Figure 2.**
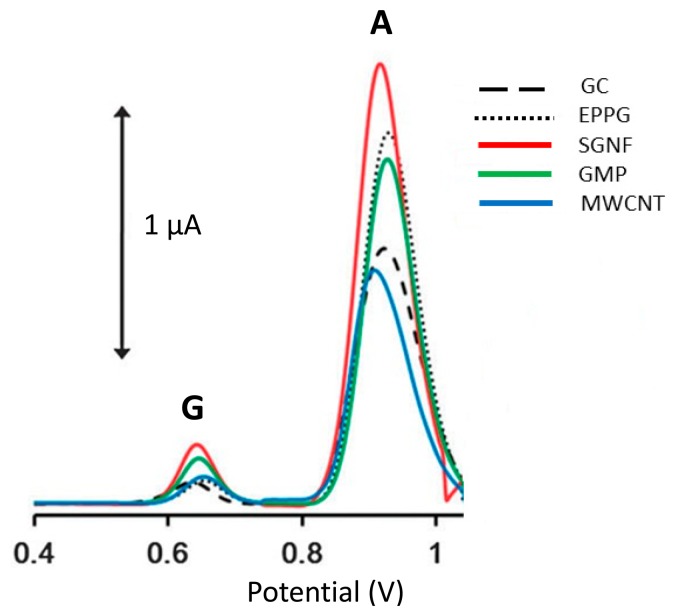
DPV of Gua (G) and Ade (A) on glassy carbon (GC), edge plane pyrolytic graphite (EPPG), stacked graphene nanofibers (SGNF), graphite microparticles (GμP), and MWCNT electrodes for electrochemical detection. Reproduced from [93] with permission.

As shown in Figure 2, the graphene based electrode enhanced two to four times oxidation of signals compared to CNTS based electrode. The 2D sheet of aromatic ring is not infinite and differs according to its basal and edge planes [96]. However, the edge plane of graphene sheets exhibits electrochemically active sites, whereas the basal plane is considered to be electrochemically inert [90]. Subsequently, significant contributions have been made on graphene-based electroanalysis of oxidations of purines [97–101]. Zhu *et al.* [102] demonstrated graphene-based electrochemical determination of Ade and Gua on a modified glassy carbon electrode which not only exhibits excellent electrochemical activity for Ade and Gua bases, but also shows high selectivity, reproducibility and stability. The detection limits (based on S/N of 3) for Ade and Gua are 0.1 μM and 0.2 μM, which are more sensitive than a graphene/Nafion composite film modified glassy carbon electrode where the detection limits of Ade and Gua were determined to be 0.75 μM and 0.58 μM, respectively [100]. The enhanced electrochemical activity contributed to the exclusive properties of graphene including large surface area, electronic properties [103].

More recently, Liu *et al.* [104] demonstrated that the polymer/graphene composite displayed excellent electrocatalytic activities for the determination of Ade and Gua nucleobases. Furthermore, Wang *et al.* [105] showed that increased porosity and acidic groups on the graphene surface enhanced the electrochemical detection of purines. The enhanced performance features of graphene, modified graphene, may be the result of increased edge plane defective sites, which enhances the electron transport [106].

#### 4.1.2. Carbon Nanotubes

**Figure 3.**
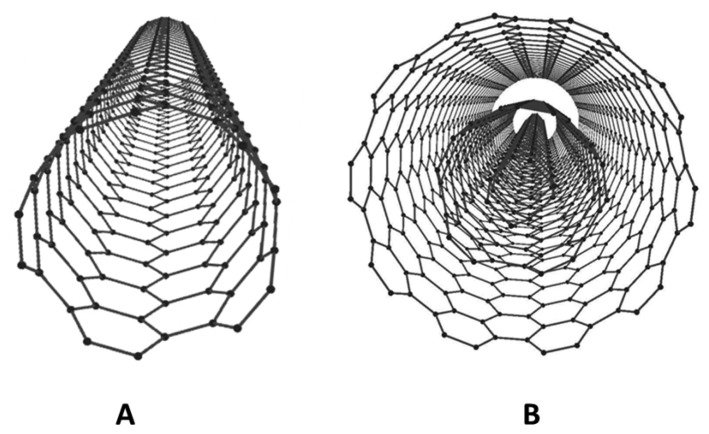
Schematic representation of (**A**) single-walled carbon nanotubes (SWCNT) and (**B**) multi-walled carbon nanotubes (MWCNT).

Carbon nanotubes (CNTs) have been extensively exploited in the area of electrochemical bio-sensing [107,108]. Since the discovery of CNTs by Iijima in 1991 [109], their exceptional electrochemical properties have captivated bioanalysts to use them as electrode materials for electrochemical analysis [110,111]. CNTs comprise single or multiple layers of needle-like cylindrical hollow structures of carbon having high aspect ratios [109]. A schematic view of single-walled carbon nanotube (SWCNT) or multi-walled carbon nanotube (MWCNT) can be seen in Figure 3.

**Figure 4.**
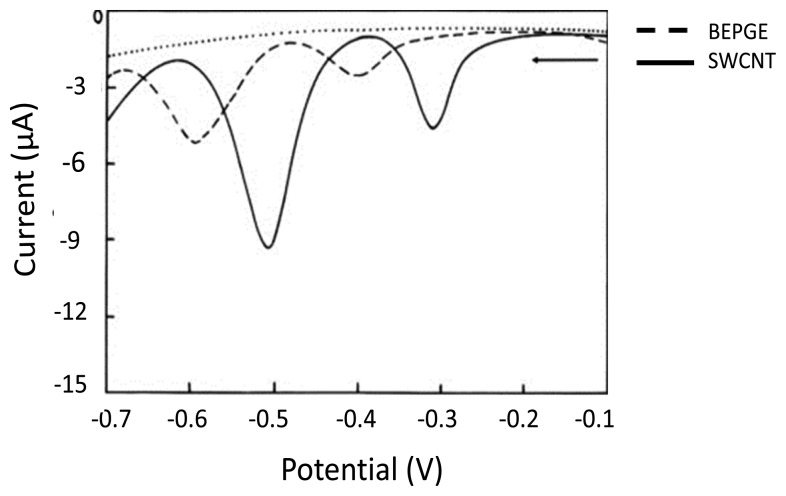
SWV of Gua and 8-hydroxyGua at (**a**) bare edge plane pyrolytic graphite electrode (BEPGE) and (**b**) SWCNT modified EPPGE; the dotted line is SWV of blank PBS using SWCNT/EPPGE at pH 7.2. Reproduced from [124] with permission.

SWCNTs are nanometer diameter wrapped sheets of sp^2^ bonded graphene [112], whereas MWCNTs are multiple rolled assemblies of SWCNT [113]. Extensive excellent reviews discuss the potential application of CNTs in the area of electrochemical detection of biomolecules due to their active participation in accelerating electron transfer [110,111,114–119]. During the last decade significant efforts have been made in using CNT-based electrochemical biosensing. The simplest strategy to fabricate CNT modified electrodes is the treatment of the electrode by a dispersion of CNT followed by evaporation of the solvent. CNT modified electrodes are highly sensitive and suitable to detect nucleobases [120]. Wu *et al.* demonstrated in 2003 [121] the dramatically enhanced response of Gua and Ade on CNT-modified GCE compared to the bare GCE surface. CNT allows a significant adsorption of nucleobases and apparently enhances the oxidation signals [121]. Recent studies have also demonstrated involvement of CNT to enhance the oxidation signals for electrochemical detection of nucleobases [122,123]. Goyal *et al.* [124] demonstrated an inexpensive and efficient tool for oxidative DNA damage detection. They observed enhanced oxidation signals of 8-hydroxyGua and Gua on a single-walled carbon nanotube (SWCNT) modified edge plane PyGE compared to the bare edge plane PyGE as shown in Figure 4. The observed limits of detection were 0.05 × 10^−9^ and 0.01 × 10^−9^ mol/L, respectively [124].

Furthermore, in another study they determined 2, 8-dihydroxyadenine (2,8-DHA) in order to monitor DNA damage by using an SMNT-modified edge plane pyrolytic graphite electrode. An enhanced electrochemical response with a limit of detection of Ade is 0.038 nM [125]. The extraordinary capability of CNT towards oxidation signals acts as an enhancer to invoke electrochemical detection of Gua and 8-hydroxyGua (Figure 4). It was demonstrated by Goyal and Bishnoi [125]. For reasons of clarity the determination of purine derivatives at various modified electrodes is summarized in Table 4.

#### 4.1.3. Nanoparticles

Nanomaterials have attracted considerable attention during the last decade in various kinds of analytical methods because of their intriguing physicochemical properties different from those of bulk materials. Particles referred to as nanoparticles (NPs), usually in the range between 1 and 100 nm, which exhibit a high surface-to-volume ratio, are potential tools for various applications in biosensing [126–128]. A vast variety of metallic [129,130] and polymeric NPs [131] have been used for electrochemical biosensing. In addition, NPs of noble metals, including gold (Au) NPs, possess potential applications in the field of biosensing due to their attractive physicochemical and catalytic properties [132,133]. Detailed information can be found elsewhere [134]. NPs play a significant role in amplifying the electrochemical response. Because of their high surface energy, NPs become unstable and aggregate, and thus it is difficult to attain monodispersity. To overcome this situation NPs are capped by any suitable stabilizing agent in order to improve the stability and performance [135]. However, due to the high energy and high surface area, NPs bind to biomolecules strongly and allow them to immobilize on the surface by electrostatic or covalent interaction for various biosensor applications [129,136]. Gold NPs have been used more frequently because of biocompatibility and stability; also other NPs were used including Ag, TiO_2_ Pt, *etc.* Oxidation of DNA bases highlights the first straightforward detection of DNA damage. Goyal *et al.* [137] used a gold NPs-modified electrode for the voltammetric determination of Guo in neutral medium. They observed an effective catalytic response towards its oxidation and estimation in the human plasma samples with good reproducibility.

The achieved LOD was 0.98 μM. Later, Goyal *et al.* [55] demonstrated a fullerene-C_60_ modified GCE for the concurrent determination of Gua and Ade. The LODs achieved were 0.14 μM and 0.3 μM, respectively. The observed detection limits were rather high. However, Xiao *et al.* [186] used a novel composite film by electrochemically coupling Au NPs on OMIMPF_6_-MWCNT film coated GCE for the simultaneous determination of Gua and Ade. The LODs achieved were 0.005 μM for both nucleobases.

Furthermore, Fan *et al.* [99] demonstrated a titanium oxide TiO_2_/graphene nanocomposite for electrochemical sensing of purine nucleobases–Ade and Gua. The NPs and graphene together enhanced the electrochemical activity and voltammetric response and the LODs achieved were 0.10 and 0.15 μM for Ade and Gua, respectively. The TiO_2_/graphene composite plays an essential role for enhancing the electrochemical activity of purine nucleobases. The enhanced voltammetric oxidation signals by TiO_2_/graphene nanocomposite significantly facilitate the electron transport and provoke the electrochemical signals of Ade and Gua [99]. El-Said *et al.* [138] developed an electrochemical sensor for the electrochemical detection of Ade and Gua and an excellent electrocatalytic activity was accomplished with a LOD of Ade (500 nM) and Gua (250 nM). Recently, Li *et al.* fabricated an electrochemical biosensor by electropolymerizing Ag ions and melamine monomer on the GCE surface (Ag-PMel/GCE) for the simultaneous determination of Ade and Gua. The electrochemical oxidative behavior of both purines was investigated by LSV and SWV. The achieved LODs were 8 nM for Ade and Gua [139]. Nanosilver possesses important characteristics as a metal nanomaterial, including the unique property of accelerating electron transport and biocompatibility, which allow electrostatic electron transfer with the electrode.

### 4.2. Self-Assembled Monolayers (SAMs)

Spontaneously arranged molecular assemblies of organic molecules in liquid phase or gas phase onto solid surfaces form self-assembled monolayers (SAMs). This provides a surface modification in a convenient and flexible way by readily lowering the surface-free energy of the substrate [187]. The molecules involved to form SAMs possess a distinct affinity with the substrate. The molecules adsorbed on the surface consist of a head group which facilitates interaction with the substrate. A broadly studied class of SAMs comes from adsorption of alkanethiols on gold [188,189]. Sowerby *et al.* [190] demonstrated a self-assembled monolayer of Ade and Gua on graphite.

Zou *et al.* [23] developed a voltammetric sensor for Ade and Gua by using GCE modified with tetraoxocalix[2]arene[2]triazine and observed a detection limit for Gua equal to 0.08 μM, whereas Jeevagan *et al.* [191] modified the GCE surface by using SAMs of 1,8,15,22-tetra-aminophthalocyanatonickel(II) (4α-Ni^II^TAPc) and achieved an enhanced electrocatalytic activity with a detection limit of 0.03 μM. The SAMs enhance the oxidation signals of Gua by enhancing its oxidation current in contrast to the bare electrode.

### 4.3. Metallic Ions

**Figure 5.**
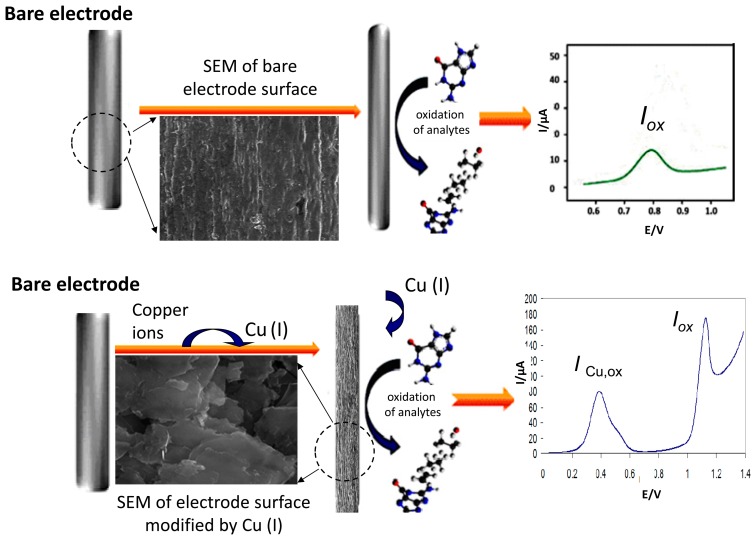
Voltammetric responses of oxidation processes of Cu(I)-purine complexes and their corresponding purines on pencil graphite electrodes (PeGEs).

When copper (II) is reduced in the presence of some purine derivatives to copper (I), it can react with these ligands forming a complex compound. The product of this electroreduction has limited stability but can be accumulated on the electrode surface. By changing the electrode potential in the positive direction the Cu (I) is oxidized back to Cu (II), thus giving rise to an oxidation voltammetric peak reflecting the presence of copper in the solution. When the purine derivative studied provides an oxidation signal in the absence of Cu (II) and then this signal is simultaneously enhanced in the presence of Cu (II), thus reflecting the amount of purine derivatives in the solution. The former peak appears at a less positive potential (*ca.* 0.4 V in neutral or slightly acidic medium) due to the dissolution of the Cu (I)-purine complex being formed at the electrode at a sufficient starting potential and accumulation time, the latter peak being located close to the original oxidation signal of analyzed purine (Figure 5).

**Figure 6.**
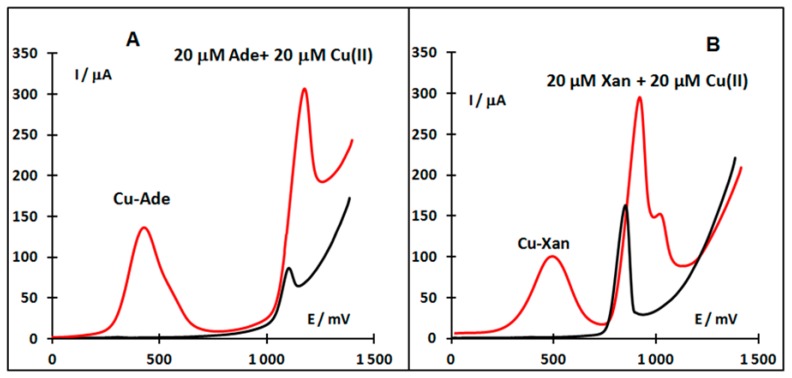
Linear sweep voltammograms of Ade (**A**) and Xan (**B**) in the absence (black line) or presence (red line) of Cu(II) ions. Acetate buffer pH 5.1; scan rate 800 mV/s; c_Ade_ = 20 μM, c_Xan_ = 20 μM, c_Cu (II)_ = 20 μM.

In the case of Xan, an electrochemical anodic stripping procedure for ultra-trace assay in the presence of Cu(II) at a glassy carbon electrode (GCE) was described [58]. The Cu (I)-Xan compound accumulated on the GCE was again dissolved by the oxidation of Cu (I) to Cu (II) and the concentration of Xan in the vicinity of the GCE increased. The results enabled us to use the measurement of the oxidation peak current as the basis of a simple, accurate and rapid method of Xan determination [58]. Differences between voltammetric signals of Ade and Xan depending on the absence and presence of Cu (II) are shown in Figure 6.

Our approach in electrochemical analysis of purine derivatives in the presence of copper is frequently based on the adsorptive stripping voltammetric (AdSV) technique in connection with elimination voltammetry with linear scan (EVLS) [192–196]. It has been known that AdSV enables measurements in a low concentration of analytes using its enhancement at the electrode by adsorptive forces. On the other hand, EVLS is a data-processing technique, which enables the elimination of selected partial voltammetric currents and the conservation of another one contributing to the increase of current sensitivity, expansion of the electrode potential range (potential window), and separation of overlapped voltammetric signals. The basic idea of the elimination procedure is based on the different dependencies of various voltammetric current components on the scan rate. The elimination result can be achieved by a function obtained by linear combination of total voltammetric currents measured at different scan rates [192–196].

In our previous papers we showed that analysis of purine nucleobases and their derivatives is possible at different carbon electrodes. We proved that on a paraffin-impregnated graphite electrode (PIGE) in chloride or bromide solutions (pH 6), the redox process of Cu(II) proceeds with two cathodic and two anodic potentially separated signals [197]. According to the elimination function E4, eliminating the kinetic and capacitive current components and conserving the diffuse current component provides the possibility of increasing current sensitivity and changing peaks into well-readable peak-counterpeaks [197]. It was suggested that the first cathodic peak corresponds to the reduction Cu(II) + e^−^ → Cu(I) with the possibility of fast disproportionation 2Cu(I) → Cu(II) + Cu(0). The E4 of the second cathodic peak signalized an electrode process controlled by a surface reaction. Anodic stripping voltammetry (ASV) on PIGE was carried out at potentials where the reduction of copper ions took place and Cu(I)-purine complexes were formed. By using ASV and CSV in combination with EVLS, the sensitivity of Cu(I)-purine complex detection was enhanced relative to either ASV or CSV alone, resulting in higher peak currents of more than one order of magnitude. Our results showed that EVLS in connection with the stripping procedure is useful for both qualitative and quantitative microanalysis of purine derivatives and can also reveal details of the electrode processes studied [197].

On the other hand, for electrochemical analysis of aminopurines (Ade, 2-aminopurine, 2,6-diaminopurine) and their complexes with Cu(I) we used a pencil graphite electrode (PeGE). The anodic process of the sparingly soluble Cu(I)-aminopurine complex, corresponding to the oxidation of Cu(I) to Cu(II), takes place in the potential range between 0.4 and 0.5 V. At more positive potentials the aminopurines provide voltammetric peaks in the order 2,6-diaminopurine (∼0.85 V), 2-aminopurine (∼1.0 V), and adenine (∼1.10 V), resulting from the oxidation of the purine ring. The appropriate complex of Cu(I)-aminopurine has a synergic effect on the height of these peaks. The stability of the accumulated complex layer was investigated by the adsorptive transfer stripping technique. The results showed that PeGE in connection with EVLS can be an excellent prototype for a cheap and fast-working sensor for aminopurines in the presence of copper [198].

Quite recently, we analyzed at the same electrode voltammetric responses of Xan and its N-methyl derivatives (1-mXan, 3-mXan, 7-m Xan, 9-mXan) in the presence of Cu(II) ions. For electroanalytical monitoring, linear sweep voltammetry (LSV) and cyclic voltammetry (CV) in connection with the EVLS were used. All substances are oxidized on PeGE providing oxidation peaks in the absence of Cu(II) between 0.83 V (Xan) and 1.08 V (7-mXan). Electrochemically produced Cu(I) ions give rise to new oxidation peaks corresponding to the oxidation of the Cu(I)-purine complex at less positive potentials between 0.32 V (9-mXan) and 0.41 V (Xan), while the former oxidation purine peaks are significantly increased. Peak-counterpeak EVLS signals reflect the irreversible charge transfer processes proceeding in an adsorbed state, and this fact confirms the suggested mechanism of electrode processes. The effect of scan rate was studied in order to evaluate the nature of the oxidation peak and to estimate kinetic parameters such as the charge transfer coefficient and the heterogeneous rate constant. The position of the methyl group on the Xan skeleton is responsible not only for changes of oxidation peak height and peak potentials but also for the mechanism of the corresponding oxidation processes [199].

PeGE was also used for voltammetric study of the 6-benzylaminopurine (BAP)/Cu(I) system. On the case of BAP, which is an adenine-type cytokinin that elicits cell division in plants and possesses antitumor activity when forming complexes with different transition metal ions as Cu(I) [200], it was shown that electrochemical analysis in connection with EVLS allows to propose the stoichiometry of the possible complexes formed and the mechanism for total electrode reactions of the BAP/Cu(I) system.

Ade detection was also performed at a nanostructured copper electrode prepared by electroplating in a porous membrane and characterized by several other methods including scanning electron microscopy, energy-dispersive X-ray spectroscopy, and electrochemical impedance spectroscopy. The research on the nanostructured copper electrode contributes to the development of advanced biosensors, which could be an environmentally safe alternative to mercury for the detection of adenine by voltammetric techniques [201].

## 5. Conclusions and Future Perspectives

Achieving high sensitivity, selectivity and cost effectiveness has always been the primary goal to design biosensing methods. Electroanalytical methods are simple, sensitive, fast, cost-effective, reliable, and efficient compared to other analytical methods. As we have summarized in this review, the integration of surface modification has opened up new possibilities in the field of electrochemical biosensing.

**Figure 7.**
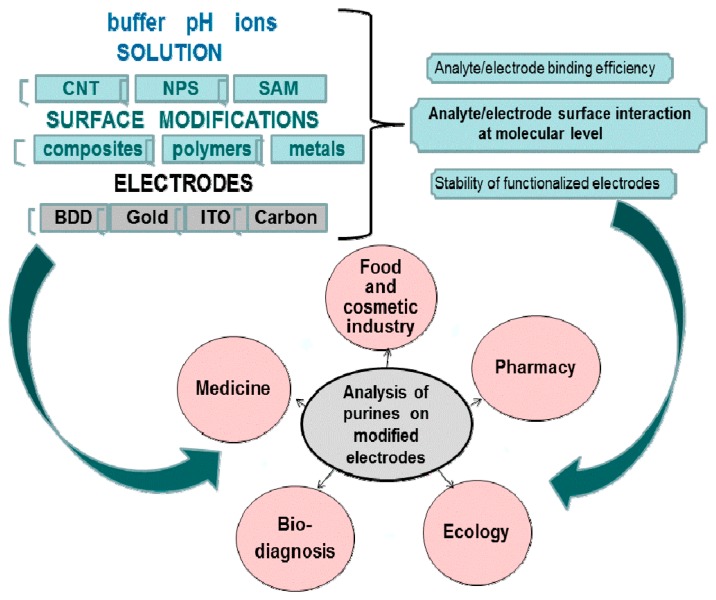
Application of electrochemical sensing of purine nucleobases and their analogues.

Figure 7 shows the range of diversities in electrochemical methods starting from buffer solution, different types of electrode with surface modifications, and indicates a very sophisticated system which needs to be more characterized at each level.

Nanomaterials provide an outstanding advantage over other types of modifications due to their outstanding electrochemical properties. From many papers it follows that any sensitive detection of purine derivatives is related to structure and chemistry on the electrode surface. It is desirable to know how the molecules are arranged on electrode surfaces and how these molecules interact with the electrode surface, *i.e.*, how they are distributed and whether the interaction between analytes and functionalized solid electrodes is strong enough to provide stability. Moreover, we need more efforts to develop an understanding of what happens at the molecular level during analyte/electrode surface interaction. Besides the range of electrodes, carbon electrodes have been used most extensively and also carbon nanomaterials have been used for modification. The critical point is besides the evident differences between carbon nanomaterials (SWCNTs, MWCNTs and graphene); the results obtained from different experiments apparently cause confusion and problems in designing an efficient electrochemical system for biosensing. There is a need, in order to develop a robust biosensing approach, to focus and concentrate on the characterization of carbon materials with different carbon nanomaterial functionalization. Obviously, the complications arisen from heterogeneous carbon nanomaterials will undoubtedly advance our understanding and therefore will increase our understanding to develop possible applications in the field of biosensing.

## Figures and Tables

**Table 1.** Adenine and adenosine.

**Electrode**	**Modification**	**Range of Linear Concentration Dependence**	**Detection Limit**	**Method**	**Application**	**Ref**
**ADENINE**						
GCE	azocalix[4]arene film	0.125–200.0 μM	70 nM	CV, DPV	detection of DNA	[18]
BDD	no modification	0.12 to 25 μM	19 nM	CV, DPV	detection of Ade in urine	[19]
PeGE	5-amino-2-mercapto-1,3,4,-thiazole	DNA concentration 50 μg/mL	-	DPV, EIS	analysis of DNA	[20]
GCE	polymerized film of melamine	0.1–60 μM	70 nM	LSV, SWV	Ade determination	[21]
CPE	DNA	-	1 ppm	CV, DPV	interaction of flutamide with DNA	[22]
GCE	tetraoxocalix[2]arene[2]tri azine	0.5–10 μM	200 nM	CV, DPV, EIS	analysis of ssDNA	[23]
GCE	1-phenyl-3-methyl-4-(2-furoyl)-5-pyrazolone	0.5–100 μM	125 nM	-	determination of Ade in pharmaceutical products	[24]
GCE	poly(sulfosalicylic acid) and ssDNA	65–1100 nM	22 nM	CV, DPV, EIS	analysis of nucleobases	[25]
PyGE	no modification	-	-	CV	redox mechanism of Ade	[11]
PeGE	-	-	-	DPV	DNA interaction with Pd and Pt complexes	[26]
carbon SPE	no modification	-	-	DPV	application in DNA genotoxicity screening	[27]
GCE	CdS-chitosan	0.02–5 μM	40 nM	DPV	analysis of damage DNA	[28]
GCE	poly-(amidosulfonic acid)	30–1000 nM	8 nM	CV, DPV, EIS	analysis of vitamin B4 tablets	[29]
GCE	cysteinic acid	0.2–100 μM	50 nM	DPV	analysis of vitamin B4 tablets	[30]
SAE, PtE, AuE, GCE	-	-	-	-	construction of electrochemical multisensors	[31]
GCE	Ade	-	40 ng/mL	SWV	detection of antioxidant capacity in flavored water samples	[32]
GCE, CPE, AuE	no modification	5–30 ng/mL	-	SWV	analysis of DNA	[33]
carbon SPE	no modification	-	-	CV, DPV	DNA damage	[34]
PeGE	no modification	-	-	AdSCP	detection of apurinic side in DNA	[35]
SPE	dsDNA	-	-	DPV	interaction of antitumor drugs with dsDNA	[36]
GCE	no modification	-	70 nM	DPV	determination of all bases in ssDNA	[5]
CPE, PeGE	-	-	-	DPV	interaction of DNA with lycorine	[37]
BDDE	no modification	0.1–8 μg/mL	3.7 ng/mL	CV, SWV	determination of ssDNA and dsDNA	[38]
GCE	no modification	-	-	CV, DPV	interaction between dsDNA and 1,2-dimyristoyl-sn-glycero-3-phosphocholine	[39]
AuE	thrombin aptamer	10–1000 nM	10 nM	DPV	analysis of Ado in human plasma	[40]
CPE	1-ethyl-3-methylimidazolium ethylsulfate	1.0–270 μM	137 nM	DPV	analysis of Ado in urine	[41]
carbon SPE	no modification	-	-	DPV	application in DNA genotoxicity screening	[27]
CPE, CILE	N-hexylpyridinium-hexafluorophosphate	1.0–140 μM	0.91 μM	DPV	Ado in human urine	[42]
PeGE	no modification	-	-	AdSCP	detection of apurinic side in DNA	[35]
GCE, PyGE	no modification	-	-	CV	oxidation of Ado	[15]

**Table 2.** Guanine and guanosine.

**Electrode**	**Modification**	**Range of Linear Concentration Dependence**	**Detection Limit**	**Method**	**Application**	**Ref**
**GUANINE**						
GCE	azocalix[4]arene film	2.5–650.0 μM	50 nM	CV, DPV	detection of DNA	[18]
BDD	no modification	0.21 to 23 μM	37 nM	CV, DPV	detection of Gua in urine	[19]
PeGE	-	-	2.92 pM in 100 mL	DPV	detection of hypermethylation of the glutathione S-transferase P1	[43]
GCE	polymerized film of melamine	0.1–50 μM	80 nM	LSV, SWV	Gua determination	[21]
CPE	DNA	-	1 ppm	CV, DPV	interaction of flutamide with DNA	[22]
GCE	tetraoxocalix[2]arene[2]tri azine	-	80 nM	CV, DPV, EIS	analysis of ssDNA	[23]
GCE	poly(sulfosalicylic acid) and ssDNA	65–1100 nM	22 nM	CV, DPV, EIS	analysis of nucleobases	[25]
PyGE, HOPG	-	-	-	CV	electrode reaction mechanism of Gua	[17]
PeGE	-	-	-	DPV	DNA interaction with Pd and Pt complexes	[26]
carbon SPE	no modification	-	-	DPV	application in DNA genotoxicity screening	[27]
GCE	CdS-chitosan	0.001–1.6 μM	2 nM	DPV	analysis of damage DNA	[28]
SAE, PtE, AuE, GCE	-	-	-	-	construction of electrochemical multisensors	[31]
PeGE	fish sperm dsDNA	0.018–2.56 ppm	-	DPV	interaction of Efavirenz with fish sperm dsDNA	[44]
GCE	Gua	-	35 ng/mL	SWV	detection of antioxidant capacity in flavored water samples	[32]
GCE, CPE, AuE	-	5–30 ng/mL	-	SWV	analysis of DNA	[33]
PeGE	fish sperm dsDNA	-	0.36 ng/mL	SWV	quantification of DNA in plant extracts	[45]
GCE	-	-	-	CV, NPV, DPV, SWV	characterization of interactions of dsDNA with a drug,	[46]
carbon SPE	no modification	-	-	CV, DPV	DNA damage	[34]
SPE	modification by tetraethoxysilane	0.19–10.8 mg/mL	0.1 μg/mL	CV, DPV	electrocatalytic oxidation of Gua	[47]
AuE	-	-	-	DPV	DNA analysis	[48]
SPE	dsDNA, ssDNA	-	-	DPV, SWV	bioanalysis of environmental pollution and DNA-drug interaction	[49]
PeGE	no modification	-	-	AdSCP	detection of apurinic side in DNA	[35]
PeGE	-	-	-		allele-specific DNA biosensor	[50]
SPE	dsDNA	-	-	DPV	interaction of some antitumor drugs with dsDNA	[36]
BDDE	-	50 μM	-		detection of nucleosides, nucleotides and oligonucleotides	[51]
AuE	-	50 μM	-	CV	guA oxidation at polycrystalline gold electrodes	[52]
GCE	no modification	-	70 nM	DPV	determination of all bases in ssDNA	[5]
CPE, PGE	-	-	-	DPV, PSA	interaction of arsenic trioxide with DNA	[53]
BDDE	no modification	0.1–8 mg/mL	10 ng/mL	CV, SWV	determination of ssDNA and dsDNA	[38]
AuE	L-cysteine monolayer	2.5–15 pmol	-	SWV	DNA hybridization	[54]
CPE, PeGE	-	-	-	DPV	interaction of DNA with lycorine	[37]
**GUANOSINE**						
GCE	no modification	-	-	CV, DPV	interaction between dsDNA and 1,2-dimyristoyl-sn-glycero-3-phosphocholine	[39]
CPE	1-ethyl-3-methylimidazolium ethylsulfate	1.0–160 μM	183 nM	DPV	analysis of Guo in urine	[41]
GCE	no modification	0.5 μM–1.0 mM	145 nM	DPV	Guo in human blood plasma and urine	[55]
BDDE	-	100 μM	-	-	detection of nucleosides, nucleotides and oligonucleotides	[51]

**Table 3.** Xanthine and hypoxanthine.

**Electrode**	**Modification**	**Range of Linear Concentration Dependence**	**Detection Limit**	**Method**	**Application**	**Ref**
**XANTHINE**						
GCE	poly L-methionine	-	4 nM	CV, DPV	detection of xanthine in serum	[59]
GCE	poly(xylitol)	1.3–75.3 μM	0.75 μM	CV	determination in urine	[60]
AuE	xanthine oxidase	2–16 μM	0.15 μM	-	xanthine in fish, chicken, pork, and beef meat	[61]
GCE	graphitized mesoporous carbon	20–320 μM	388 nM	-	human blood-plasma, urine and fish samples	[62]
PtE	polyvinylchloride	25–400 nM	25 nM	-	xanthine in fish meat, cow and buffalo milk	[63]
GCE	3-aminopropyltriethoxysilane/glutaraldehyde/xanthine oxidase/chitosan	0.5–18 μM	21.5 nM	-	construction of biosensors, determination of xanthine	[64]
GCE	mesoporous carbon	20–200 μM	46 nM	CV, DPV	determination of xanthine	[65]
GCE	polymerized film of bromocresol purple	0.1–100 μM	60 nM	DPV	simultaneous determination in human serum	[66]
GCE	acetylene black and dihexadecyl hydrogen phosphate	three orders of magnitude	60 nM	CV	rat striatal microdialysates of freely moving rats	[67]
**HYPOXANTHINE**						
GCE	poly L-methionine	-	8 nM	CV, DPV	detection of hypoxanthine in serum	[59]
GCE	poly(xylitol)	5–55 μM	4.5 μM	CV	determination in urine	[60]
GCE	graphitized mesoporous carbon	20–240 μM	351 nM	DPV	human blood-plasma, urine and fish samples	[62]
GCE	3-amino-5-mercapto-1,2,4-triazole	-	-	DPV	hypoxanthine in human blood serum and urine samples	[68]
GCE	mesoporous carbon	20–150 μM	69 nM	CV, DPV	determination of hypoxanthine	[65]
GCE	polymerized film of bromocresol purple	0.2–80 μM	120 nM	DPV	simultaneous determination in human serum	[66]
GCE	acetylene black and dihexadecyl hydrogen phosphate	three orders of magnitude	250 nM	CV	rat striatal microdialysates of freely moving rats	[67]
**INOSINE**						
GCE	3-amino-5-mercapto-1,2,4-triazole	-	50 nM	DPV	Inosine in human blood serum and urine samples	[68]

**Table 4.** Determination of purine derivatives on modified electrodes.

**Electrode**	**Modification**	**Range of Linear Concentration Dependence**	**Detection Limit**	**Method**	**Application**	**Ref**
**ADENINE**						
ITO	gold nanodots	0.25–90 μM	500 nM	CV, DPV	Ade determination in human serum	[138]
GCE	Ag-PMel	0.1–60 μM	8 nM	CV, SWV, LSV	detection of Ade	[139]
GCE	chitosan carbon nanofibers	0.2–50 μM	73.8 nM	CV	detection of beef kidney sample	[140]
Pt	MWCNT	-	3 μM	LSV	detection of Ade	[141]
Pt	Au NPs/graphene	-	-	LSV	detection of Ade	[142]
GCE	Au nps	-	4 nM	CV, DPV	detection of Ade	[143]
CPE	TiO_2_-NPs-MgY/Zm	-	0.02 μM	CV, DPV,	detection of Ade	[144]
GCE	graphite nanopowder and MWCNT	-	-	DPV	detection of Ade in meat	[145]
GCE	reduced graphene oxide	-	200 nM	-	detection of Ade in ssDNA	[102]
GCE	reduced graphene	20 μM oligonucleotides	-	DPV	analysis of DNA	[146]
GCE	reduced graphene oxide	units to hundreds mM	-	DPV	detection of DNA	[147]
GCE	multi-layer of graphene	units to tens of mM	-	-	construction of genosensors	[148]
GCE	boron-doped carbon nanotubes	-	-	DPV	detection of DNA in the field of genetic-disease diagnosis	[70]
SPE	β-cyclodextrin/poly(N-acetylaniline)/CNT	10–1020 μM	50 nM	-	sensor of DNA hybridization	[149]
GCE	MWCNT/choline	-	-	-	simultaneous detection of DNA	[122]
GCE	ssDNA and AuNP	-	-	CV, DPV	inhibition effect of Ade	[150]
GCE	PbO_2_/CNT/1-butyl-3-methylimidazolium hexafluorophosphate	-	30 nM	-	analysis of herring sperm DNA	[151]
GCE	different chemically-modified graphene	-	-	DPV	device for label-free DNA analysis	[152]
GCE	TiO_2_-graphene nanocomposite	0.5–200 μM	0.1 μM	CV, DPV	electrochemical sensor of Ade	[99]
PyGE	SWCNT	5–100 nM	3.7 nM	SWV	Ade in human urine	[153]
GCE	TiO_2_ nanobelts	-	-	CV, DPV	analysis of DNA	[154]
GCE	MWCNT	-	80 nM	LSV, CV	analysis of DNA	[155]
GCE	CdS-chitosan	-	nM range	DPV	analysis of DNA damage	[28]
graphene NF	CNT	-	-	-	analysis of DNA	[93]
GCE	DNA/MWCNT	-	-	CV, DPV, SWV	monitoring of phenolic pollutants	[156]
GCE, AuE, ITO	MWCNT/poly(new fuchsin)	-	-	CV, DPV		[84]
GCE	β-cyclodextrin/MWCNT	-	0.75 nM	DPV		[157]
GCE	MWCNT, AuNP	0.008–2.0 μM	5 nM	CV,	analysis of milk, plasma and urine	[158]
GCE	Ade	-	40 ng/mL	SWV	detection of antioxidant capacity in flavored water samples	[32]
GCE	CNTs/La(OH)_3_	-	220 nM	CV, DPV	analysis of DNA	[159]
GCE, CPE, AuE	-	5–30 ng/mL	-	SWV	analysis of DNA	[33]
GCE, AuE, TOE	MWCNT, AuNP, hydroxypropyl-β-cyclodextrin	-	-	CV, DPV	electrochemical characterizations and surface morphology studies	[160]
GCE	SWCNT/poly(acridine orange)	-	1.8 nM	CV, DPV	simultaneous determination of Ade in DNA	[161]
PyGE	β-cyclodextrin/CNT	-	0.2 mg/mL	DPV	simultaneous or individual determination of Ade	[120]
SPE	MWCNT	-	-	CV	detection DNA and RNA	[162]
GCE	SWCNT	-	-	CV, DPV	analysis of DNA	[163]
GCE	SWCNT	-	-	CV, DPV	analysis of DNA	[164]
**ADENOSINE**						
PyGE	SWCNT	10–100 nM	7.6 nM	SWV	Ado in human urine	[153]
GCE	fullerene C_60_	0.01–100 μM	80 nM		Ado in urine	[12]
GCE	fullerene C_60_	0.5–1000 μM	302 nM	DPV	Ado in urine and blood plasma	[165]
GCE	fullerene C_60_	0.5 μM–1.0 mM	302 nM	DPV	Ado in human blood plasma and urine	[55]
**GUANINE**						
ITO	gold nanodots	-	250 nM	DPV	Ade determination in human serum	[138]
GCE	Ag-PMel	0.1–50 μM	8 nM	CV, SWV, LSV	detection of Gua	[139]
GCE	chitosan carbon nanofibers	0.2–50 μM	46.8 nM	CV	detection of beef kidney sample	[140]
GCE	graphene oxide	-	-	CV, DPV	determination of Gua	[166]
GCE	Au NPS	-	5 nM	CV, DPV	detection of Gua	[143]
CPE	TiO_2_nps-MgY/Zm	-	0.013 μm	CV, DPV,	detection of gua	[144]
GCE	MWCNT/ionic liquid/chitosan	0.5–30 nM	37 pM	DPV	detection of human immunoglobulin E	[167]
PeGE	-	-	2.92 pM in 100 mL	DPV	detection of hypermethylation of the glutathione S-transferase P1	[43]
CPE	nanostructured Pt thin film	0.1–500 μM	31 nM	DPV	determination of Gua	[168]
GCE	reduced graphene	20 μM oligonucleotides	-	DPV	analysis of DNA	[146]
GCE	reduced graphene oxide	-	150 nM	-	detection of Gua in ssDNA	[102]
GCE	boron-doped carbon nanotubes	-	-	DPV	detection of DNA in the field of genetic-disease diagnosis	[70]
SPE	β-cyclodextrin/poly(N-acetylaniline)/CNT	10–1020 μM	50 nM	-	sensor of DNA hybridization	[149]
GCE	reduced graphene oxide	units to hundreds mM	-	DPV	detection of DNA	[147]
GCE	multi-layer of graphene	units to tens of mM	-	-	construction of genosensors	[148]
PeGE	polymer-ZnO nanoparticle	-		DPV, EIS	sequence-selective DNA hybridization	[169]
GCE	MWCNT/choline	-	-	-	simultaneous detection of DNA	[122]
GCE	PbO_2_/CNT/1-butyl-3-methylimidazolium hexafluorophosphate	-	6 nM	-	analysis of herring sperm DNA	[151]
GCE	worm-like cobalt oxide nanostructures	40 nM–10 μM	3 nM	CV	determination of Gua	[170]
GCE	different chemically-modified graphene	-	-	DPV	device for label-free DNA analysis	[152]
GCE	poly(sulfosalicylic acid) and ssDNA	65–1100 nM	22 nM	EIS	analysis of nucleobases	[25]
GCE	TiO_2_-graphene nanocomposite	0.5–200 μM	0.15 μM	CV, DPV	electrochemical sensor of Ade	[99]
GCE	TiO_2_ nanobelts	-	-	CV, DPV	analysis of DNA	[154]
CPE,	SWCNT/cobalt phthalocyanine	-	130 nM	CV, DPV	detection of DNA hybridization	[171]
GCE	MWCNT	-	20 nM	LSV, CV	analysis of DNA	[155]
PeGE	SnO_2_ NPS-poly (vinylferrocenium)	-	-	DPV	DNA hybridization	[172]
PyGE, HOPG	-	-	-	CV	electrode reaction mechanism of Gua	[17]
PeGE	-	-	-	DPV	DNA interaction with Pd and Pt complexes	[26]
PyGE	SWCNT	-	-	0.17 nM	simultaneous determination of Gua and 8-hydroxyguanine	[124]
GCE	CdS-chitosan	-	nM range	DPV	analysis of DNA damage	[28]
graphene NF	CNT	-	-	-	analysis of DNA	[93]
GCE	SWCNT	40–110 nM	3 nM	-	DNA hybridization biosensor	[173]
GCE	MWCNT	-	-	-	Gua in human prostate cancer (PC-3) cell suspension	[174]
GCE	DNA/MWCNT	-	-	CV, DPV, SWV	monitoring of phenolic pollutants	[156]
GCE, AuE, ITO	MWCNT/poly(neofuchsin)	-	-	CV, DPV	-	[84]
GCE	β-cyclodextrin/MWCNT	-	0.75 nM	DPV	-	[157]
SAE, PtE, AuE, GCE	-	-	-	-	construction of electrochemical multisensors	[31]
PeGE	SWCNT	-	-	DPV, EIS	interaction between daunorubicin and dsDNA	[175]
GCE	MWCNT, Au NPS	0.008–2.0 μM	5 nM	CV	analysis of milk, plasma and urine	[158]
CPE	molybdenum (VI) complex-TiO_2_ nanoparticle	7.0–200 nM	3.4 nM	CV, DPV, CPA, CHC	detection of Gua at nanomolar levels	[176]
GCE	CNTs/La(OH)_3_	-	260 nM	CV, DPV	analysis of DNA	[159]
GCE, AuE, TOE	MWCNT, Au NPS, hydroxypropyl-β-cyclodextrin	-	-	CV, DPV	electrochemical characterizations and surface morphology studies	[160]
GCE	SWCNT/poly(acridine orange)	-	0.9 nM	CV, DPV	simultaneous determination of Ade in DNA	[161]
PyGE	β-cyclodextrin/CNT	-	0.1 mg/mL	DPV	simultaneous or individual determination of Ade	[120]
GCE	Carbon_60_	0.5–100 μM	60 nM	CV	determination of Gua	[165]
SPE	MWCNT	-	-	CV	detection DNA and RNA	[162]
GCE	SWCNT	-	-	CV, DPV	analysis of DNA	[163]
nanoelectrode arrays	MWCNT nanoelectrode arrays embedded in an SiO_2_ matrix	-	-	-	disposable chips for rapid molecular analysis based on the Gua oxidation	[177]
GCE	SWCNT	-	-	CV, DPV	analysis of DNA	[164]
nanoelectrode arrays	MWCNT nanoelectrode arrays embedded in an SiO_2_ matrix	-	-	-	detection of DNA PCR amplicons	[178]
**XANTHINE**						
GCE	luteolin/MWCNT	-	0.65 μM	CV, DPV	detection of xanthine	[179]
GCE	MWCNT	0.2–10 μM	0.1 μM	CV	detection of xanthine	[180]
GCE	PAP/RGO	-	0.5μM	CV	detection of xanthine	[181]
GCE	poly (pyrocatechol violet)/carboxyl MWCNT	-	500 nM	DPV	human serum samples	[40]
ultra-thin CPE	SWCNT/poly(azure I)	0.2–100 μM	600 nM	CV	human urine samples	[182]
GCE	MWCNT, SWCNT	200 μM	134 nM	DPV	fish samples	[183]
**HYPOXANTHINE**						
GCE	reduced graphene oxide attached through 1,6-hexadiamine	-	320 nM	DPV	human blood plasma and urine	[184]
GCE	poly(L-arginine)/graphene composite film	0.20–20 μM	10 nM	DPV	hypoxan in human urine	[185]
GCE	poly (pyrocatechol violet)/carboxyl MWCNT	-	20 nM	DPV	human serum samples	[40]
ultra-thin CPE	SWCNT/poly(azure I)	0.4–100 μM	28 nM	CV	human urine samples	[182]
GCE	MWCNT, SWCNT	150 μM	2.87 μM	DPV	fish samples	[183]

## Abbreviations

Ade: adenine
Ado: adenosine
AMP: adenosine monophosphate
AuE: gold electrode
BAP: 6-benzylaminopurine
BDD: boron doped diamond
BDDE: boron-doped diamond electrode
CILE: carbon ionic liquid electrode
CNT: carbon nanotubes
CV: cyclic voltammetry
DPV: differential pulse voltammetry
EIS: electrochemical impedance spectroscopy
GCE: glassy carbon electrode
Gua: guanine
Guo: guanosine
Ino: inosine
ITO: indium tin oxide
LOD: limit of detection
MWCNT: multi-walled carbon nanotubes
NPs: nanoparticles
ODN: oligodeoxynucleotide
PeGE: pencil graphite electrode
PtE: platinum electrode
PyGE: pyrolytic graphite electrode
SAE: solid amalgam electrode
SAM: self-assembled monolayer
SPE: screen-printed electrode
SQV: square-wave voltammetry
SWCNT: single-walled carbon nanotubes
Xan: xanthine
Xao: xanthosine
